# Teleburn: Designing A Telemedicine Application to Improve Burn Treatment

**DOI:** 10.2174/1874431101812010033

**Published:** 2018-08-31

**Authors:** Farhang Hosseini, Haleh Ayatollahi, Seyed Hamid Salehi, Kazemzadeh Jafar

**Affiliations:** 1 Department of Health Information Management, School of Health Management and Information Sciences, Iran University of Medical Sciences, Tehran, Iran; 2 Iran University of Medical Sciences, Tehran, Iran; 3 Urmia University of Medical Sciences, Tehran, Iran

**Keywords:** Telemedicine, Teleconsultation, Burn, Medical informatics, Dermatology, WHO, QUIS

## Abstract

**Background:**

Due to the increasing rate of the burn injuries and a limited number of specialized treatment centers, providing medical advice and medical care at the point of need is necessary. The aim of the present study was to design and implement a teleburn system to enhance the quality of care for the burn patients.

**Methods:**

This study was completed in 2016. In order to design the system, information needs assessment was conducted by using a questionnaire. The participants of this phase were five specialists, five general practitioners, and 12 nurses. The setting of the study was the burn department of a public hospital and a burn center. The prototype of the system was designed based on the findings derived from the first phase, and the usability of the system was evaluated later.

**Results:**

The teleburn system was a web-based system with different sections for GPs/nurses and specialists. In total, 28 burn consultations were made successfully by using the system. The findings of the usability testing showed that most of the participants evaluated the system at a good level. The mean score for the specialists, general practitioners and nurses was 8.4±0.46, 7.7±0.39, and 7.5±0.51, respectively.

**Conclusion:**

Although it was the first time in the country that the teleburn system was designed and introduced to the clinicians, they seemed to be satisfied with using the system. This system could help general practitioners and nurses to receive specialist's advice on a timely manner to improve the treatment of the burn patients. However, more research should be conducted to determine the effectiveness of using this technology in the real work environment.

## 1 INTRODUCTION

Telemedicine technology has made medical interventions possible even even when physicians and patients are far away from each other [1, 2]. Telemedicine is defined as the electronic exchange of medical data between two locations [3-5]. World Health Organization (WHO) defines telemedicine as follows: “the delivery of healthcare services, where distance is a critical factor, by all health care professionals using information and communication technologies for the exchange of valid information for diagnosis, treatment and prevention of disease and injuries, research and evaluation, and for the continuing education of health care providers, all in the interests of advancing the health of individuals and their communities” [6]. The use of telemedicine technology has a number of advantages, such as accelerating the process of care, improving clinical diagnoses and treatments and reducing unnecessary patients’ transportation between health care centers. Besides, telemedicine can enhance the knowledge and skills of general practitioners by providing a learning environment and exchanging knowledge between GPs and specialists [3, 4].

One of the applications of telemedicine is treating the burn patients [3, 6]. The findings of the related studies show that due to the geographical dispersion and a limited number of specialized burn centers, the use of telemedicine for the burn patients is vital and can help to reduce medical errors significantly when treating these patients [3, 7-9]. Although wound assessment is associated with some visual factors, such as the size and color of the wound and some subjective factors, such as pain and itching, the use of digital images and video conferencing helps to improve the accuracy of diagnoses and work efficiency [4, 10-12].

On the other hand, caring for the burn patients is of crucial importance especially during the first 24 to 48 hours of accident. However, the lack of experience in some nurses and general practitioners, who provide primary care for the burn patients, might be harmful to the patients and may affect their care plan [6, 13]. As a result, the use of technology, such as telemedicine has been suggested to be able to contact specialists and provide the best care plan for the burn patients during 24 hours a day, seven days a week [14]. This type of technology, which is also called teleburn, allows patients to receive required treatments at the medical centers in their hometown, and helps to identify patients who need to be referred to the burn centers [15-21]. Moreover, general practitioners and nurses can be trained in t burn care and wound care using this technology [15-17].

Another reason for using telemedicine technology in different medical areas, such as burn is to improve health equity and access to the medical services. Although providing equitable access to medical services is one of the important goals in different countries [16, 22, 23], literature review shows that providing necessary facilities for burn patients is not possible in most countries, including IRAN [18, 22]. In IRAN, most of the facilities and burn centers are concentrated in certain areas mainly due to the lack of required equipment, geographical distribution of cities, and the limited number of specialists in the burn specialty. For example, there is only one burn center in the eastern IRAN [23]. In addition, insufficient hospitals’ spaces, expensive medical equipment, low interest in working at burn centers, and limited funds are other problems which need to be taken into account [24]. Therefore, the purpose of this study was to design and implement a teleburn system to improve healthcare delivery for the burn patients in the country.

## 2 MATERIALS AND METHODS

This study was conducted in 2016 and consisted of two main phases. Initially, essential data elements and required features for the system were determined. In the second phase, a web-based teleburn system was developed based on the findings derived from the first phase, and its usability was evaluated. The settings of the study were a burn department of a public hospital and a burn center. The public hospital was located in one of the North West cities of the country and had 19 units including a burn unit with 27 beds. The hospital was equipped with an intranet, a high-speed internet network, and computers in all medical units. Burn patients were referred to the burn unit of this hospital from other cities, and were hospitalized or referred to the burn centers in neighboring provinces. The burn center was located in the capital and had 113 beds. About 30 to 40 percent of patients from other cities were referred to this hospital.

In the first phase, general practitioners (n=3) and nurses (n=12) who worked in the burn unit of the public hospital, and plastic surgeons (n=5) who worked in the burn center participated in the study. In the second phase, a total of 13 system users (including five plastic surgeons, three general practitioners, and five nurses) evaluated the usability of the system. The participants were different from the participants of the first phase. Essential data elements and system features were determined by distributing a two-choice questionnaire which was designed based on the literature and common forms used in the burn centers [25-30] and consisted of 72 questions in three sections: personal information of patients and physicians, essential data elements, and necessary features of the system. Two choices were “necessary” and “unnecessary” for each question.

Face and content validity of the questionnaire was approved by five nurses, general and plastic surgeons who worked in the settings of the study. The reliability of the questionnaire was measured by calculating KR-20 (α=0.78).

The usability of the system was evaluated by using the think-aloud method [31, 32] and a standard questionnaire, the Questionnaire for User Interaction Satisfaction (QUIS), version 5.5 [33, 34]. It was a 10-point Likert scale questionnaire and contained five sections (27 questions). These sections are as follows: overall reaction to the software (six questions), screen design and layout (four questions), terminology and systems information (six questions), learnability (six questions), and system features (five questions). The reliability of the questionnaire was confirmed in the previous study (α = 0.94) [33].

To design the system, the data were analyzed from the first phase and a rule was set by the researchers. According to this rule, all data elements and system features with at least 60 percent agreement among the participants were considered essential and were included in the system and those with lower level of agreement were not included. After developing the system, a number of participants (n=13) were asked to use the system and express their opinions about the system usability. In total, 28 burn consultations were made successfully by using the system and the identity of patients and clinicians remained confidential during data collection. The data derived from the usability evaluation were also analyzed and the results were reported at three levels: weak (0-3), Medium (3.1-6), and good (6.1-9). Mean values were calculated for different parts of the standard questionnaire and were reported separately for general practitioners, specialists and nurses.

## 3 RESULTS

A total of 20 clinicians (five surgeons, three general practitioners and 12 nurses) participated in the first phase of the study. Most nurses (n=5; 41.7%) and all surgeons (n=5; 100%) aged between 41 to 50 years and were male. Most general practitioners aged between 31 to 40 years (n =2; 66.7%) and were male (n =2; 66.7%).

### 3.1 Data Elements and System Features

The results showed that most of the questionnaire's items were recognized “necessary” by at least 60 percent of the participants. For example, all items related to burn, such as burn degree, Total Burn Surface Area (TBSA), and burn severity were recognized “necessary” to be included in the system. However, patient data, such as address, the name of workplace, place of birth, marital status, father’s name, and education level were found “unnecessary” by the majority of the participants. Table **1** demonstrates required data elements and system features which were found “necessary” by at least 60% of the participants.

The prototype of the system was designed by using PHP programming language and based on the results derived from the first phase of the study. MySQL database management system was used to create a highly functional and interactive web site which could be accessible for multiple users and helped to store, retrieve, search and organize data. The system was designed for the use of specialists and GPs/nurses, and all data must be entered manually. During the study, no connection was established between this system and the other health information systems, such as patients’ hospital records. The first page of the system included the login screen with a general description of the system and related regulations, along with some information about the burn scientific association in IRAN and hospitals participating in this research. New users could enter the system by clicking on the registration form and by entering the required information, such as name, surname, date of birth, gender, phone number, email address, username and password. Also, the user type (specialist, GP, nurse) was determined while completing the registration form. Then, the system administrator approved the form and the user could access to the relevant section.

### 3.2 Features for GPs and Nurses

**Fig. (1):**
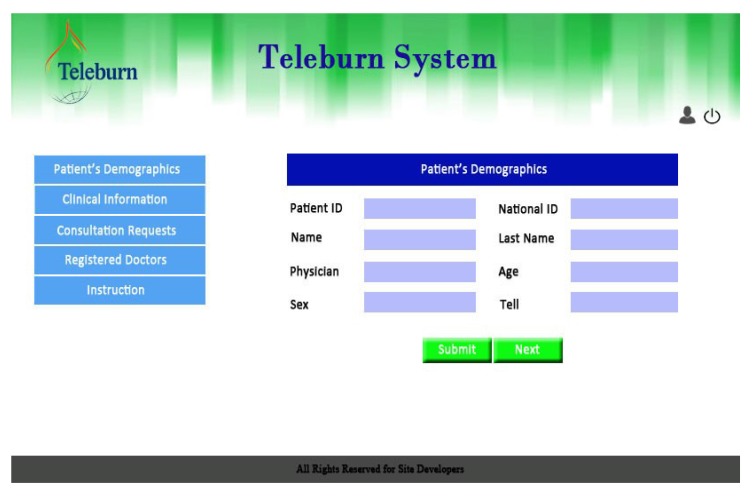
Teleburn's features for GPs and nurses.

The final version of the system included five links for GPs and nurses as follows: patient registration, patient's clinical information, consultation requests, a list of registered specialists, and instructions about how to use the system (Fig. **1**).

**Fig. (2):**
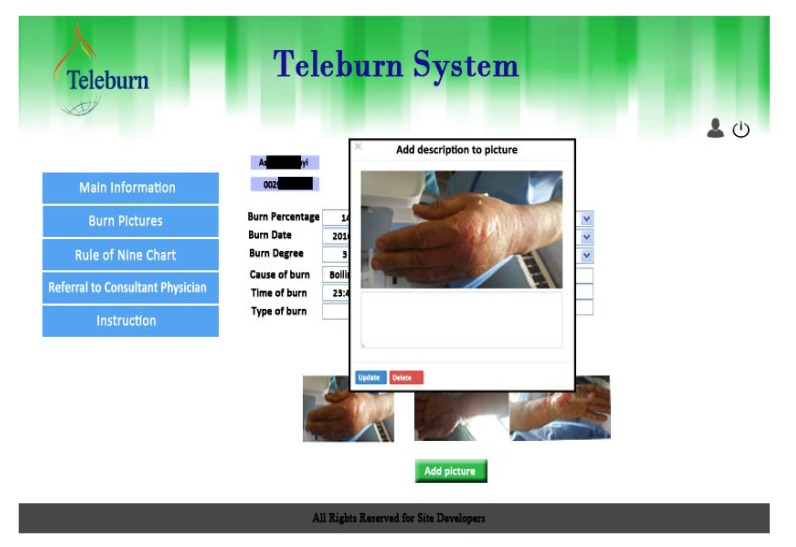
Consultation request in the Teleburn system.

After completing patient's demographic information, clinical data (burn information) and the results of medical examinations were recorded. Then, photos and videos were taken and uploaded to the system. In the next step, a rule of nines diagram could be painted by clicking on the image of the patient's body and the burn percentage could be automatically calculated based on the burn data entered into the system previously. Finally, the specialist’s consultation request was submitted (Fig. **2**).

### 3.3 Features for Specialists

Three links for specialists included viewing consultation requests and patient information submitted by the GPs or nurses, responding to the consultation requests along with giving medical advice, and instructions about how to use the system. Specialists could see a list of consultation requests by logging into the system. By clicking on the patient’s name, the specialist could see more information about the patient clinical status. Then, they could respond to the consultation request and send it back. A specialist was informed about the consultation request by receiving an email and a Short Message Service (SMS). As a result, responding to the consultations could be on time.

### 3.4 Usability Testing

In this study, the prototype of the system was run on a personal computer in both public hospital and the burn center. Initialy, one of the researchers introduced the system to five participants (including three GPs and two specialists) and asked them to express what they see, do, or feel while working with the system. During each session, participants' performance and comments were recorded by the researcher. Voice recording was used in all sessions and each session lasted about 20 minutes. Participants’ comments were used to improve the final version of the system.

The second approach to evaluate the usability of the system was using a standard questionnaire, namely Questionnaire for User Interaction Satisfaction (QUIS) version 5.5. To do this, the initial version of the system was provided to 13 users (including five specialists, three general practitioners, and five nurses). In total, 28 burn consultations were made successfully by using the system. Afterwards, the users were asked to complete the questionnaire and express their comments and feelings about the system. Table **2** shows the results of the evaluation study.

As Table **2** shows, the mean values reported for GPs, specialists, and nurses were between 7 and 9 for different parts of the questionnaire. The highest mean value for GPs (8.33 ± 0.38) was related to the system terminology and information; for nurses (7.95 ± 0.43), it belonged to the screen design and layout, and for specialists (8.67 ± 0.44), it was related to the overall reaction to the software. The lowest mean value for GPs (7.28 ± 0.38) was related to overall reaction to the software; for nurses (7.16 ± 0.54), it belonged to the terminology and system information, and for specialists (7.88 ± 0.61), it was related to the system features. Overall, the results suggested that the users evaluated the usability of the system at a good level.

## 4 DISCUSSION

To date, different systems and applications have been developed in different countries to improve the quality of care for burn patients [10]. The findings of several studies revealed that telemedicine applications are one of the solutions to improve burn care, and in some cases, it can be considered as an alternative to face-to-face examinations [10, 21, 35]. For example, Lo *et al*. showed that using tele-education courses in burns can significantly improve the quality of care, decrease anxiety in burn patients, increase the knowledge of physicians and patients, and reduce stress and tensions [36]. In other studies, the use of imaging technology in the field of burn care and telemedicine has been highlighted [27, 28, 37]. It seems that using internet and web-based systems along with imaging technology have a crucial impact on improving care services [38-42]. Such a technology can also be useful for developing countries, like IRAN, in which the number of burn centers is very limited and the specialists and plastic surgeons mainly work in the large cities [27, 43, 34].

In the present study, a teleburn system was developed. It was the first time in the country that such an application was designed and implemented. The findings indicated that most of the proposed data elements and system features were recognized as necessary by the system users (specialists, general practitioners, and nurses). In a similar study, Turk *et al*. implemented a web-based teleburn system to facilitate communication and information exchange. By using this system, GPs were able to communicate with specialists, and they could improve their experiences *vial* this type of communications [6]. However, in the present study, the system was developed based on the users' requirements, and patient information was collected in detail. Such a system could also be considered as an electronic medical record for the burn patients. Moreover, displaying a rule of nines diagram was considered for the system to calculate the burn percentage automatically.

In the present study, the usability of the system was evaluated by the real users. The results indicated that the majority of users were satisfied with the system and the overall reaction was positive indicating the acceptance of the system at an early stage. Similarly, in 2012, Halt *et al*. conducted a study to investigate users’ satisfaction with the use of telemedicine among burn centers in the United States. According to their findings, 38 out of 126 burn centers used telemedicine applications with the method of store and forward and about 80% of patients and health care providers were satisfied with the use of telemedicine applications in the burn centers [12]. The results of other studies also show the high level of users' satisfaction with the use of telemedicine applications in the burn centers [2, 6, 10, 37]. Therefore, it can be concluded that the results of the current study are in line with the findings of similar studies.

Overall, the findings revealed that telemedicine applications can be used in the burn centers using minimum effort and infrastructure. As the first 24 to 48 hours of an accident is vital for a burn patient and due to the lack of expertise in providing a suitable care plan for the burn patient by nurses or GPs, telemedicine applications can be used to enhance quality of care for this group of patients [6, 13]. Such a system can help to overcome geographical dispersion and establish a connection with specialists at the early stage of assessment, diagnosis and management of the burn patients.

## 5 LIMITATIONS

As noted before, it was the first time in IRAN that the teleburn system was designed and implemented to improve the quality of care for the burn patients. Although a limited number of participants (specialists, GPs and nurses) participated in this study, the aim was developing a new telemedicine application rather than generalizing the results to a larger population. Therefore, the researchers believe that the limited number of participants does not affect the design of the system. Moreover, in this study, an initial version of the teleburn system was developed mainly due to the time and resources restrictions. The system can be improved in the future, for example by adding more features, comprehensive care plans and protocols, and a calorie calculator. In terms of the usability testing, only the usability of the initial version of the system was investigated in the current study. Therefore, conducting evaluation studies on a larger scale with more participants and in a real working environment are suggested.

## CONCLUSION

The burn-related injuries need to receive prompt attention at the very early stage of an accident to be able to reduce the consequences. To accelerate patient care and to overcome the geographical dispersion and the limited number of burn centers, the use of information and communication technology has been suggested. The teleburn system developed in this research was an example of telemedicine applications that could be easily used to access the specialists in a timely manner. The designed system was simple and user-friendly and the necessary burn data, such as the rule of nines diagram were included in the system. However, more features can be added to the system in the future to improve its usability. In addition, the clinical effectiveness of the system was not examined in this research. Therefore, conducting pre- and post-implementation studies can be beneficial to identify the effectiveness of the system.

## Figures and Tables

**Table 1:** Required data elements and system features for teleburn.

Data Elements	Patient’s demographic data	First name, last name, age, sex, height, weight and contact number
	Patient’s clinical data	Vital signs, allergies and other drug reaction, current drugs, vaccination, time of burn, cause of burn, type of burn, date of burn, medical history of patient
System Features	Images	Ability to load images, remove images, add description to the images, rotate images, zoom in and out, edit images, send images
	Automation	Automatic calculation of TBSA, paint the rule of nines diagram, fluid resuscitation level, input data control
	Reporting	Statistical reporting (number of referrals, consultations, treatments, patients and doctors), Reporting based on time, date, name, national ID, insurance ID

**Table 2:** Usability of the teleburn system from the users' perspectives.

Participants Evaluation Areas	General Physicians Mean ±SD	Nurses Mean ±SD	Specialists Mean ±SD
Overall reaction to the software	7.28 ± 0.38	7.17 ± 0.52	8.67 ± 0.44
Screen design and layout	7.58 ± 0.43	7.95 ± 0.43	8.6 ± 0.24
Terminology and system information	8.33 ± 0.38	7.16 ± 0.54	8.5 ± 0.44
Learnability	7.61 ± 0.29	7.63 ± 0.54	8.16 ± 0.56
System features	7.93 ± 0.46	7.44 ± 0.52	7.88 ± 0.61
